# Salvage Retzius sparing robotic assisted radical prostatectomy: the first brazilian experience

**DOI:** 10.1590/S1677-5538.IBJU.2021.0260

**Published:** 2021-06-25

**Authors:** Igor Nunes-Silva, Alexandre Kyoshi Hidaka, Carlos Roberto Monti, Marcos Tobias-Machado, Hamilton de Campos Zampolli

**Affiliations:** 1 Instituto do Câncer Dr. Arnaldo Vieira de Carvalho São PauloSP Brasil Instituto do Câncer Dr. Arnaldo Vieira de Carvalho - IAVC, São Paulo, SP, Brasil; 2 IN Cancer Center São PauloSP Brasil IN Cancer Center, São Paulo, SP, Brasil; 3 Instituto de Radium Oncology CampinasSP Brasil Instituto de Radium Oncology, Campinas, SP, Brasil; 4 Clinica Onco Hematos AracajuSE Brazil Clinica Onco Hematos, Aracaju, SE, Brazil; 5 Faculdade de Medicina do ABC Divisão de Urologia Santo AndréSP Brasil Divisão de Urologia, Faculdade de Medicina do ABC - FMABC, Santo André, SP, Brasil; 6 Pontifícia Universidade Católica de Campinas Faculdade de Medicina Divisão de Urologia CampinasSP Brasil Divisão de Urologia, Faculdade de Medicina, Pontifícia Universidade Católica de Campinas - PUC-Campinas, Campinas, SP, Brasil

## Abstract

**Introduction:**

Salvage Radical Prostatectomy after radiation therapy is challenging and associated with high rates of serious complications (1, 2). The novel Retzius-Sparing RARP (RS-RARP) approach has shown excellent continence outcomes (3, 4).

**Purpose:**

To describe step-by-step our Salvage Retzius-Sparing RARP (sRS-RARP) operative technique and report feasibility, safety and the preliminary oncological and continence outcomes in the post-radiation scenario.

**Materials and Methods:**

Twelve males presenting local prostate cancer recurrence after radiotherapy that underwent sRS-RARP were included. All patients performed preoperative multiparametric MRI and PSMA-PET. Surgical technique: 7cm peritoneum opening at Douglas pouch, Recto-prostatic space development, Seminal vesicles and vas deferens isolation and section, Extra-fascial dissection through peri-prostatic fat, Neurovascular bundle control, Bladder neck total preservation and opening, Anterior dissection at Santorini plexus plane, Apex dissection with urethra preservation and section, Prostate release, Vesicouretral modified Van Velthoveen anastomosis, Rocco Stitch, Oncological and continence outcomes reported with minimum 1-year follow-up.

**Results:**

Ten patients had previously received external beam radiation (EBR) whereas two received previous brachytherapy plus EBR. At 1, 3 and 12 months after surgery, 25%, 75% and 91.6% of the men used one safety pad or less, respectively. No major complications or blood transfusions were reported. Final pathology reported pT2b 41.6%, pT2c 33.3% and pT3a 25%, positive surgical margins 25%, positive lymph nodes were not found, biochemical recurrence 16.6%.

**Conclusion:**

Salvage Retzius-Sparing Robotic Assisted Radical Prostatectomy approach appears to be technically feasible and oncologically safe with potential to provide better continence outcomes.
